# The role of genetic polymorphisms at the chromosomes 5p15, 6p12, 6p21 and 15q25 in non-small-cell lung cancer prognosis: a Portuguese prospective study

**DOI:** 10.1186/1753-6561-7-S2-P57

**Published:** 2013-04-04

**Authors:** Ramon A de Mello, Mónica Ferreira, Filipa S Pires, Sandra Costa, Michael Luis, João Cunha, Pedro Oliveira, Venceslau Hespanhol, Rui M Reis

**Affiliations:** 1 Department of Medicine, Faculty of Medicine University of Porto, 4200-319, Porto, Portugal; 2 Department of Medical Oncology, Portuguese Oncology Institute, 4200-072, Porto, Portugal; 3 Life and Health Sciences Research Institute (ICVS), School of Health Sciences, University of Minho, Braga, Portugal; 4 3B's - PT Government Associate Laboratory, Braga/Guimarães, Portugal; 5 Department of Pneumology, Centro Hospitalar de São João, 4200-319, Porto, Portugal; 6 Department of Pneumology, Hospital de Braga, Braga, Portugal; 7 Department of Populations Studies, ICBAS, Universidade do Porto, Porto, Portugal; 8 Molecular Oncology Research Center, Barretos Cancer Hospital, Barretos-SP, Brazil

## Introduction

Genome Wide Association Study (GWAS) variants on chromosome 15q25 and 5p15 and genetic polymorphisms on the vascular endothelial growth factor (VEGF) gene showed that they may contribute to lung carcinogenesis. Therefore, this study was performed in order to assess the role of GWAS, and VEGF variants in non-small-cell lung cancer (NSCLC) prognosis.

## Materials and methods

Prospective study from February 2010 to April 2011. Median follow up was 12 months. NSCLC patient’s genotyping was performed using the Sequenom® MassARRAY platform. Kaplan-Meier curve was used to assess overall survival (OS) and progression-free-survival (PFS). Statistical significance was considered for p < 0.05.

## Results

144 NSCLC patients were consecutively genotyped in order to assess 19 single nucleotide polymorphisms (SNPs). Males were 78.5%. Median age was 61.5 (32 – 89) years-old. Non-squamous cell histology was 77.1% and 91.4% were stages IIIB and IV. The following SNPs showed influence in OS: rs2010963 (*VEGF* + 405 G/C), p = 0.042, rs3025010 (*VEGF* intron 5 C/T), p = 0.047, rs401681 C/T on 5p15, p = 0.046, rs31489 C/A on 5p15, p = 0.029; and these SNPs showed influence in PFS: rs9295740 G/A on 6p21, p = 0.074, rs401681 C/T on 5p15, p = 0.021.

## Conclusions

This was the first large study in Portugal involving NSCLC patients and assessment of 19 SNPs on chromosome 5p15.33, 6p12, 6p21, 6p21.3, and 15q25. Our study suggests that variants on chromosome 5p15 and 6p21 are prognostic biomarkers in advanced NSCLC. In the future, genome-identified patients may improve NSCLC screening strategies and therapeutic management.

## Financial support

University of Minho, FAPESP and CAPES.

